# Developing a Mental Health eClinic to Improve Access to and Quality of Mental Health Care for Young People: Using Participatory Design as Research Methodologies

**DOI:** 10.2196/jmir.9716

**Published:** 2018-05-28

**Authors:** Laura Ospina-Pinillos, Tracey A Davenport, Cristina S Ricci, Alyssa C Milton, Elizabeth M Scott, Ian B Hickie

**Affiliations:** ^1^ Brain and Mind Centre The University of Sydney Sydney Australia; ^2^ School of Medicine University of Notre Dame Sydney Australia

**Keywords:** mental health, community-based participatory research, eHealth

## Abstract

**Background:**

Each year, many young Australians aged between 16 and 25 years experience a mental health disorder, yet only a small proportion access services and even fewer receive timely and evidence-based treatments. Today, with ever-increasing access to the Internet and use of technology, the potential to provide all young people with access (24 hours a day, 7 days a week) to the support they require to improve their mental health and well-being is promising.

**Objective:**

The aim of this study was to use participatory design (PD) as research methodologies with end users (young people aged between 16 and 25 years and youth health professionals) and our research team to develop the Mental Health eClinic (a Web-based mental health clinic) to improve timely access to, and better quality, mental health care for young people across Australia.

**Methods:**

A research and development (R&D) cycle for the codesign and build of the Mental Health eClinic included several iterative PD phases: PD workshops; translation of knowledge and ideas generated during workshops to produce mockups of webpages either as hand-drawn sketches or as wireframes (simple layout of a webpage before visual design and content is added); rapid prototyping; and one-on-one consultations with end users to assess the usability of the alpha build of the Mental Health eClinic.

**Results:**

Four PD workshops were held with 28 end users (young people n=18, youth health professionals n=10) and our research team (n=8). Each PD workshop was followed by a knowledge translation session. At the conclusion of this cycle, the alpha prototype was built, and one round of one-on-one end user consultation sessions was conducted (n=6; all new participants, young people n=4, youth health professionals n=2). The R&D cycle revealed the importance of five key components for the Mental Health eClinic: a home page with a visible triage system for those requiring urgent help; a comprehensive online physical and mental health assessment; a detailed dashboard of results; a booking and videoconferencing system to enable video visits; and the generation of a personalized well-being plan that includes links to evidence-based, and health professional–recommended, apps and etools.

**Conclusions:**

The Mental Health eClinic provides health promotion, triage protocols, screening, assessment, a video visit system, the development of personalized well-being plans, and self-directed mental health support for young people. It presents a technologically advanced and clinically efficient system that can be adapted to suit a variety of settings in which there is an opportunity to connect with young people. This will enable all young people, and especially those currently not able or willing to connect with face-to-face services, to receive best practice clinical services by breaking down traditional barriers to care and making health care more personalized, accessible, affordable, and available.

## Introduction

### Background

The Internet and emerging technologies have long been identified as having the potential to significantly expand the reach of quality mental health care by addressing geographical, economical, and human resource barriers [1,2]. Over the past decade, evidence suggests that the Internet is considered not only a major source of information about health (including mental health) and well-being [3-5], but that it is also useful for mental health promotion, screening, prevention, early intervention, and referral processes [6-9]. Web-based platforms are also being used to deliver real-time stepped care services [10], providing information and low-intensity treatment for those in the early stages of help-seeking, with the capacity to increase scope and intensity of treatments as illnesses progress [11].

In Australia, new and emerging mental health technologies are urgently needed as 1 in 5 young Australians aged between 16 and 24 years experience a mental health disorder each year, yet only 1 in 4 receives professional help [12]. Of those who do receive help, only a small proportion receives timely and evidence-based treatments [13]. Access to quality care is especially difficult for disadvantaged and vulnerable populations, including children; Aboriginal and Torres Strait Islander young peoples; young people from culturally and linguistically diverse backgrounds; and young people living in regional, rural, and remote areas [14,15].

Today, with ever-increasing availability and use of technology, the potential to reach young people is especially promising [7]. For example, in 2014-15, 85% of people aged 15 years and over in Australia were Internet users, and young people in the age group of 15 to 17 years had the highest proportion of use (99%) [16]. Importantly, there is also a growing body of literature supporting the use of Internet-based treatment approaches for a range of mental health problems in adults and adolescents [6,17-19]. Although there is far less research focusing on systems that integrate real-time Web-based stepped care support in mental health services [20], some research examining comprehensive Web-based support systems is starting to emerge [21,22].

### Web-Based Health Information

Young people are increasingly relying on the Internet to find answers about their health concerns. For example, in Australia, a national survey in 2012-13 revealed that 53% of young people aged between 16 and 25 years with moderate and very high levels of psychological distress sought Web-based information related to a mental health and/or alcohol or other substance use problem; the majority found this information to be helpful [23]. In other surveys, young people reported feeling comfortable accessing Web-based mental health tools because they felt they were anonymous, welcoming, less stigmatizing, and, for the most part, trustworthy [24]. Similarly, in the United States, a survey in 2013 found that 59% of patients had gone online to look for health information in the previous year and 35% had gone online to self-diagnose or diagnose someone else’s condition. Of the “online diagnosers,” 46% concluded that they needed to see a health professional, whereas 38% preferred a self-management option [25].

Similarly, a small pilot study evaluating an online triage platform also found that, for every user requiring a general practitioner response through an e-consultation, 5 users required online self-help only (WebGP, 2014) [26,27]. The Internet, therefore, offers people seeking information with a useful and easy gateway to answers and solutions that respond to their needs.

### Internet-Based Screening

Individuals often show a preference for computerized screening over face-to-face interviews when the subject matter is sensitive in nature [28-32]. Importantly, Internet-based screening for common mental health problems has been shown to be reliable and effective [9,33,34]. In relation to screening for suicidal ideation, it is argued that the implementation of standardized, self-reported, computer-based assessments (with stringent suicide response protocols) may be a strategy that is both accurate and viable if followed up with health professional support [20,35]. In general, there is, however, the potential that symptom, Web-based assessment tools may increase demand on services as the tools are often risk averse (due to medico-legal concerns), recommending professional care when self-management is an appropriate alternative [36,37].

### Videoconferencing

The provision of mental health services though videoconference systems have been widely used since the 1960s [38]. Videoconferencing is viewed as more advantageous than telephone support as the health professional can gauge important visual cues to inform their assessment such as appearance, facial expression, motor activity, movements, and mannerisms [39]. Supportive Web-based conversations with a health professional and referral to appropriate resources have also been found to negate risky behaviors, such as suicide and violence, in highly distressed people [40]. Importalty, videoconferencing has been found to be as reliable as face-to-face assessments, and more cost-effective [41].

In Australia, telepsychiatry (videoconferencing) has been practiced since the 1990s and its benefits have been translated into a wide range of populations including rural populations, Indigenous communities [42], the defense force [43], and children and adolescents [44]. Surprisingly, despite the reliability and benefits of videoconferencing, use of videoconference systems remains low in the different settings [45]. Embedding videoconferencing in comprehensive Web-based support systems would enable a more systematic use of this technology and a greater number of people to access mental health services.

### Comprehensive Web-Based Mental Health Care

The augmentation of traditional videoconferencing with online self-reported health questionnaires or screening results has now emerged in the research literature [20,46]. In a study conducted by Williams and colleagues, participants who screened positive for major depression or suicidality were given the opportunity to schedule a videoconference via Skype with a psychiatrist within a 2-week period [46]. In a recent study conducted by our team, an initial Web-based clinical assessment (with an embedded suicidality escalation protocol) was completed as a routine part of the assessment process (either before a video or face-to-face visit with a health professional) for a subset of participants. After the assessment was completed, the severity of mental illness was determined using a clinical staging model [47], and those who were considered to be high-risk (according to the suicidality escalation protocol) were escalated to the youth health service, bringing forward their initial face-to-face appointment [20].

### Mental Health Interventions for Young People

In a systematic review of Web-based mental health interventions for young people, however, limited uptake, engagement, adherence, and dropout rates have been identified as significant problems [48,49]. Some researchers, including ourselves, believe that the involvement of young people and youth health professionals in the design, development, and delivery of youth services could lead to better engagement and outcomes [50-52].

### Participatory Design and Technology-Based Mental Health Interventions

Participatory design (PD) methodologies, which were developed in the late 1960s and early 1970s, emphasize the importance of involving all stakeholders (including end users, developers, and researchers) during the development of products to help ensure the end product meets everyone’s needs; improve usability; and increase engagement of users [53-55]. The process involves iterative design cycles in which end users and researchers contribute to knowledge production and the development of the end product [50,56]. Importantly, end users should participate in all stages of development [57], not as consultants or controllers of the process, but sharing equal responsibility with the research team for the outcomes [50]. Some researchers [58], including us, consider PD as key research methodologies that overtly put the end user at the center of research and here, the development of the MHeC [59].

The use of PD is expanding in the development of technology-based mental health and well-being interventions for young people. In 2015, a systematic review [50] described the development of these interventions in areas such as prevention, screening [60], and treatment programs [61]. In the majority of cases, however, end users assumed more of a consultative role. Despite the fact that uptake of PD in the development of technology-based mental health and well-being interventions has increased, evidence is still needed to assess the impact of these research methodologies in the outcomes of these interventions [50].

As consumers have the opportunity to share their preferences before the development of expensive, and potentially helpful, systems, the rationale behind the use of PD in the development of mental health technologies could mean that an active engagement of end users could reduce the 17-year gap in translational research [62].

### This Study

In Australia, Web-based mental health services include health promotion, self-directed, and low intensity mental health support (eg, ReachOut! [63]; beyondblue [64,65]); national online counseling services (eg, eheadspace [66]); structured self-directed online therapy (eg, MoodGYM [67]); and those offering a combination of assessment and structured online therapy, including additional therapist support (eg, MindSpot Clinic [68]).

What is missing is a Web-based mental health clinic that includes access to all the components and services necessary to meet the needs of all young people (those between the ages of 16 and 25 years), regardless of where they are on the spectrum of mental health or ill-health, or where they reside in Australia.

The aim of this study was to use PD with end users (young people and youth health professionals) and our research team to codesign and build a Mental Health eClinic (MHeC) to improve timely access to, and better quality, mental health care for young people across Australia.

## Methods

### Participants

Participants included young people attending *headspace* Camperdown and *headspace* Campbelltown, and youth health professionals from both *headspace* Camperdown and *headspace* Campbelltown (*headspace* is the national youth mental health foundation dedicated to improving the well-being of young Australians; Camperdown and Campbelltown are two different sociodemographic areas of Sydney).

The University of Sydney’s Human Research Ethics Committee approved the study (Protocol No. 2014/689). For all phases, participants (young people aged between 16 and 25 years and youth health professionals) who expressed an interest to participate in the study were provided with the participant information statement and participant consent form before providing consent and participating in the study. Parental consent was also obtained for participants aged between 16 and 17 years. At the conclusion of each PD workshop and one-on-one end user consultation, each young person was provided with a gift voucher to thank them for their participation and sharing their expertise; young people were also reimbursed for any travel-related expenses to attend the workshop or session. Youth health professionals were not provided with gift vouchers as they participated in stages 1 and 3 during work time. All workshops were catered.

### Recruitment Strategy

Recruitment strategies for young people included the posting of flyers at each *headspace* center inviting young people to be involved in the study, and informing youth health professionals and reception staff at each *headspace* center about the study so they too could assist with recruitment. All young people belonging to *headspace* Camperdown and *headspace* Campbelltown Youth Reference Group were also invited to participate in the study. Inclusion criteria for the study were as follows: young person attending either *headspace* Camperdown or *headspace* Campbelltown; aged between 16 and 25 years; and with access to the Internet through a mobile phone, tablet, desktop, or laptop.

In relation to the recruitment of youth health professionals, senior management at *headspace* Camperdown and *headspace* Campbelltown informed all staff about the study and called for expressions of interest to participate.

### Research and Development Cycle

**Figure 1:**
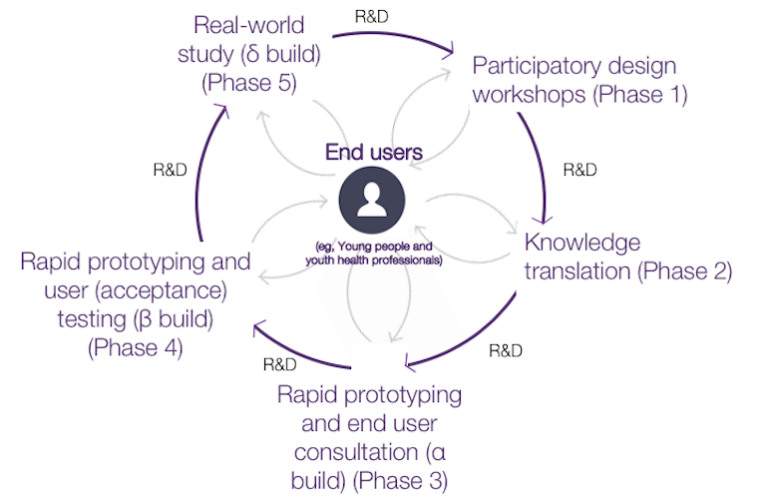
PD research methodologies used during the design and build of the MHeC where end users participate in all stages of development, are at the center of the R&D cycle, and share equal responsibility with the research team for the outcomes. PD: participatory design; MHeC: Mental Health eClinic; R&D: research and development.

The research and development (R&D) cycle for the codesign and build of the MHeC included several iterative PD phases (Figure 1): PD workshops (phase 1); translation of knowledge and ideas generated during workshops (“knowledge translation”; KT) to produce mockups of webpages (either hand-drawn sketches or wireframes; phase 2); and rapid prototyping and one-on-one consultations with end users, including assessing the usability of the online alpha build of the MHeC (phase 3). The remaining phases of PD for the MHeC (rapid prototyping and user [acceptance] testing of the beta build [phase 4], and real-world study of the delta build [phase 5]) are currently underway and will be reported separately.

### Participatory Design Workshops (Phase 1)

The PD employed in this study was informed by the research methodologies developed by the Young and Well Cooperative Research Centre for the development of evidence-based online youth mental health promotion, intervention, and treatment [58]. PD workshops were developed to accommodate a maximum of 12 participants per workshop. The same group of researchers facilitated all PD workshops. Importantly, a mental health professional was present on-site for the duration of all workshops involving young people in case anyone experienced psychological distress as a result of the subject matter. A scribe was present to take hand written notes at each workshop.

PD workshops were held in two stages with young people and youth health professionals attending separate workshops (stage 1) or a combined workshop (stage 2; Textbox 1). Following each workshop, the knowledge and ideas generated during the workshop were translated to produce mockups of webpages, either as hand-drawn sketches or wireframes produced using Balsamiq (Balsamiq Solutions, LLC, Sacramento, California, United States: a rapid wireframing tool that reproduces the experience of sketching on a whiteboard but using a computer) [69]. The mockups were then presented at the next workshop, enabling content and broad design ideas to be discussed, critically analyzed, and further developed.

**Phases 1 and 2 workflow.**

- Phase 1, stage 1 (young people only, Camperdown): One 1-day participatory design (PD) workshop with young people attending *headspace* Camperdown; workshop held in *headspace* Camperdown

Knowledge translation for young people only (phase 2)

- Phase 1, stage 1 (young people only, Campbelltown): One 1-day PD workshop with young people attending *headspace* Campbelltown; workshop held in *headspace* Campbelltown.

Knowledge translation for youth health professionals only (phase 2)

- Phase 1, stage 1 (youth health professionals only): One 1-day PD workshop with youth health professionals from *headspace* Camperdown and *headspace* Campbelltown; workshop held in *headspace* Campbelltown.

Knowledge translation combined workshop (phase 2)

- Phase 1, stage 2 (young people and youth health professionals combined): One half-day PD workshop with both young people and youth health professionals from *headspace* Camperdown and *headspace* Campbelltown (all participants must have previously participated in a stage 1 workshop); workshop held in *headspace* Camperdown.

**Figure 2:**
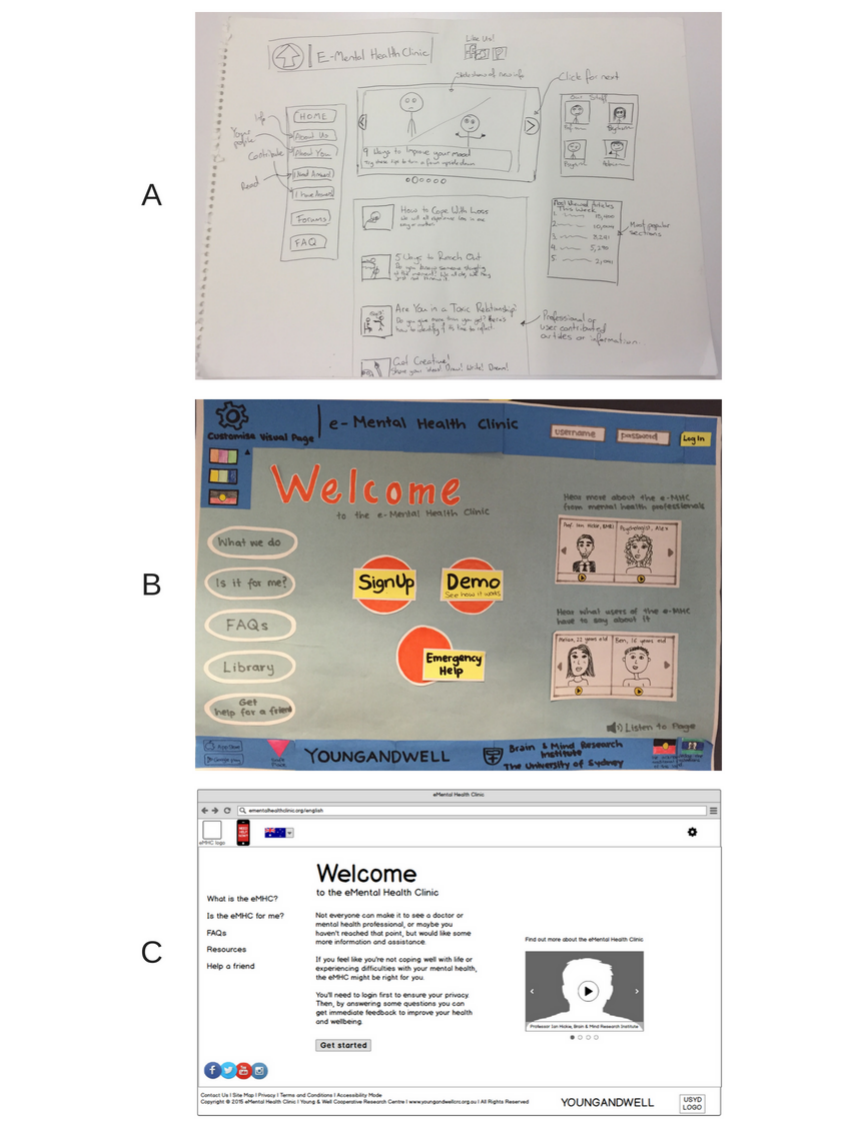
Samples of visual artefacts. (A) Hand-drawn sketch by end users during a PD workshop; (B) Hand-drawn sketch of a webpage generated following a KT session; and (C) Wireframe generated using Balsamiq following a KT session. PD: participatory design; KT: knowledge translation.

Topics for stages 1 and 2 workshops included the MHeC home page; important website functions; look and feel of the website; online physical and mental health assessment; provision of assessment results online (including consideration of a dashboard of results); a video visit system (utilizing live interactive videoconferencing); and the development of personalized well-being plans based on assessment results. Importantly, technology was not used in any PD workshop; instead, several artifacts and design activities (Figure 2) were used in each workshop to facilitate discussions and the design process. The activities included:

1. The use of propositions to explore and communicate the concept of the MHeC
2. End user sketching [70] (hand-drawn sketches by young people and youth health professionals, either individually or in groups) to enhance the feedback process and generation of new ideas
3. Analysis of mockups of webpages (hand-drawn sketches and wireframes) to test designs and provide feedback about the look, feel, content, and behavior of the MHeC.

### Knowledge Translation (Phase 2)

At the conclusion of each workshop, the transcript and all visual artifacts were independently analyzed by a KT Team (three 2nd-and 3rd-year psychology students [AI, ML, and ED], all females aged between 20 and 23 years) who were interns at the time at The University of Sydney’s Brain and Mind Centre). Observations were tallied, and those observations with three or more tallies were considered for inclusion in the next generation of wireframes for discussion and analysis at the following workshop. Information was compiled until saturation point was reached (defined as the point where no new information was attained) [71].

### One-on-One Consultations With End Users, Including Assessing the Usability of the Alpha Build of the Mental Health eClinic (Phase 3)

Phase 3 involved in-depth one-on-one consultations with new end users (young people and youth health professionals who had not participated in Phase 1). The inclusion of new participants aimed to reduce biased responses due to habituation or familiarity with the topic as a result of prior participation in the study. In each 90-min one-on-one end user consultation, a researcher was paired with a participant (end user) and an observer took notes. Sessions involved the use of laptops where participants had access to the alpha build of the MHeC website. Employing a think-aloud protocol [72], participants were observed as they navigated the MHeC and responded to questions posed by the researcher about the main components of the MHeC; responses were recorded by the observer. The initial effectiveness of the system was then assessed by asking participants to complete 3 usability tasks: (1) create an account and login; (2) find the “need help now” button; and (3) book an appointment. Task completion time was recorded to assess the efficiency of the system. No instructions or clues were provided, and comments in relation to navigation were recorded.

### Data Analysis

Qualitative data were interpreted using thematic analysis techniques [73] according to the following themes: general elements of the MHeC; general look and feel; privacy and data sharing; and interaction of the MHeC with social networks. Records of all tallies obtained in Phases 1-3 were then grouped and interpreted by a team of researchers (LOP, TD, and 2nd- and 3rd-year psychology students [AH and FY] who were interns at the time at The University of Sydney’s Brain and Mind Centre). Each theme and associated content was discussed by the group, and differences of opinion were discussed until consensus was reached.

## Results

**Figure 3:**
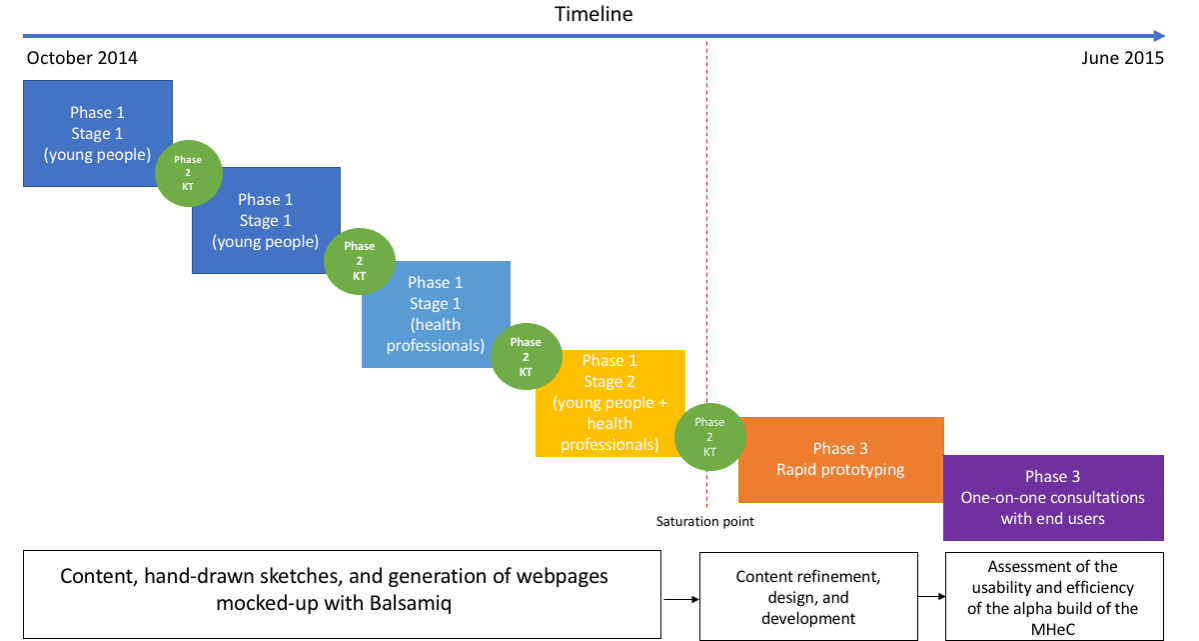
Study Gantt chart. KT: knowledge translation; MHeC: Mental Health eClinic.

A total of 4 PD workshops, 4 knowledge translation sessions, and 1 round of one-on-one end user consultation sessions were conducted between October 2014 and June 2015 (Figure 3).

### Participant Characteristics

PD workshops were held with young people attending *headspace* Camperdown (n=7) and *headspace* Campbelltown (n=11), and youth health professionals working at those services (*headspace* Camperdown n=5; *headspace* Campbelltown n=5). A total of 18 young participants participated in stage 1 (young people only) PD workshops: there was equal gender participation, and 78% (14/18) were aged between 18 and 25 years. Ten youth health professionals participated in stage 1 (youth health professionals only) PD workshop: the majority of these participants were female (70%, 7/10) and aged between 20 and 30 years (70%, 7/10). The group comprised 4 psychologists, 2 occupational therapists, 1 medical student, 1 general practitioner, 1 social worker, and 1 Aboriginal youth worker.

Nine participants participated in stage 2 (young people and youth health professionals combined): young people (n=5) and youth health professionals (n=4). The majority of the participants were male (56%, 5/9); the youth health professional’s group included 3 psychologists and 1 occupational therapist.

Six people participated in Phase 3 (consultation with end users, including usability assessment of the online alpha build). The majority of these participants were female (67%, 4/6). The group contained 1 clinical psychologist, 1 psychology student, and 4 young people.

### Main Components of the Alpha Build of the Mental Health eClinic

**Figure 4:**
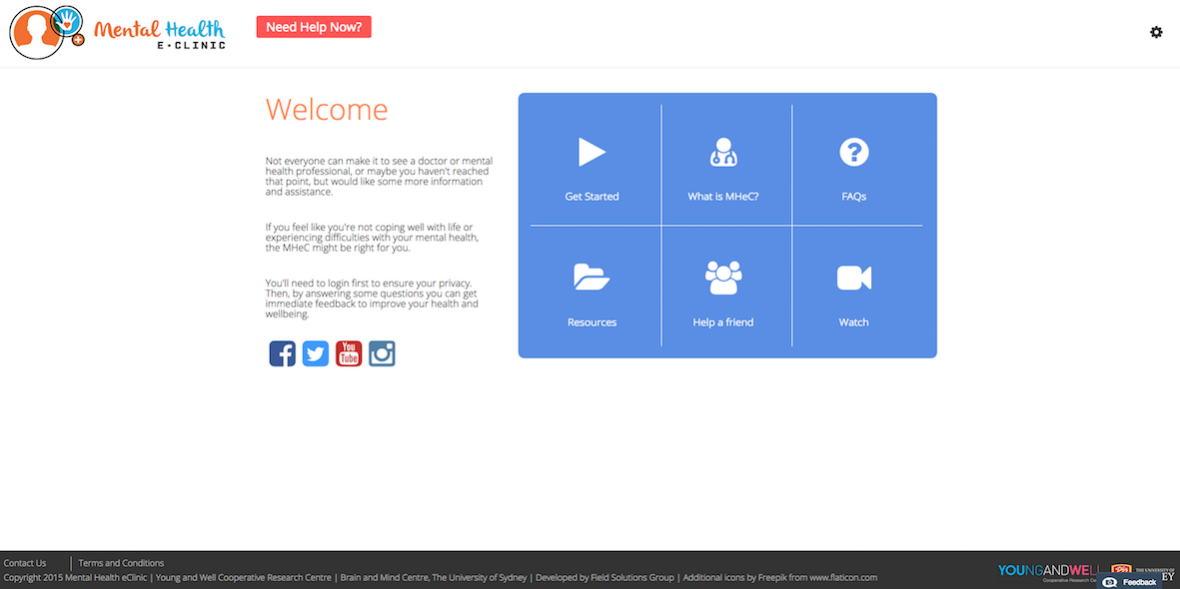
Home page with a clearly visible triage system for those requiring urgent help.

**Figure 5:**
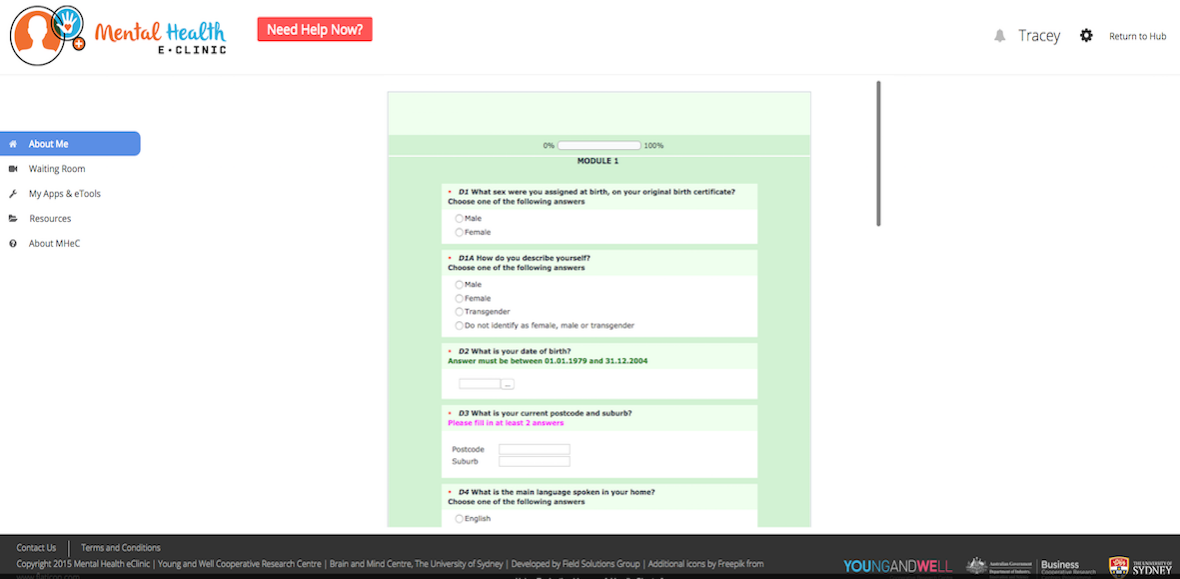
Comprehensive online physical and mental health assessment.

**Figure 6:**
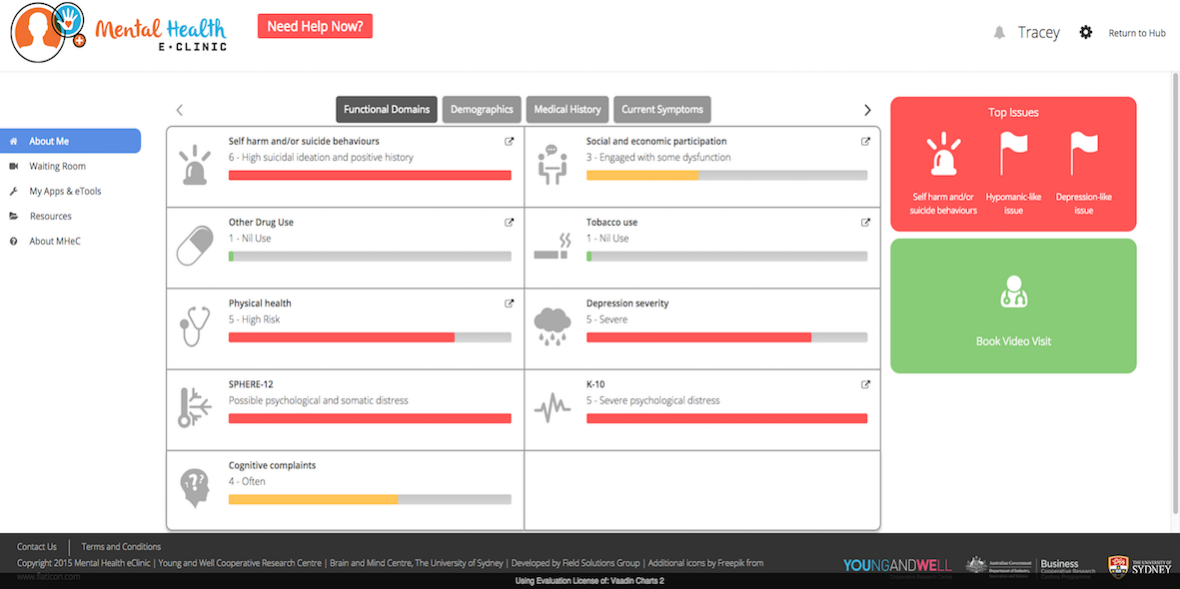
Dashboard of results and progress report.

**Figure 7:**
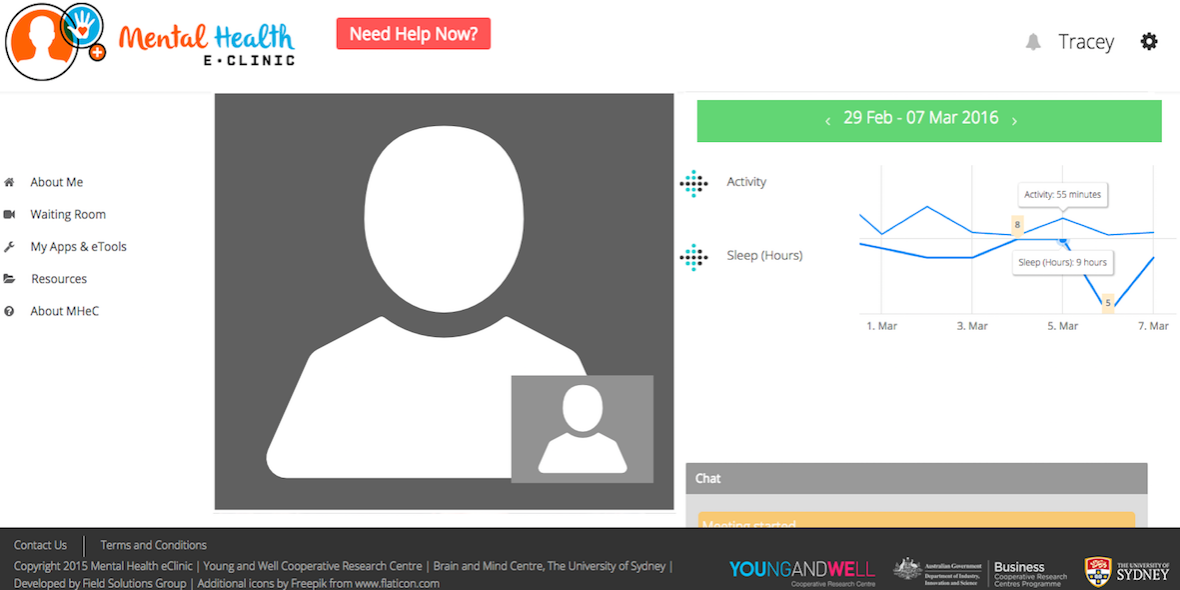
Booking and video visit system.

**Figure 8:**
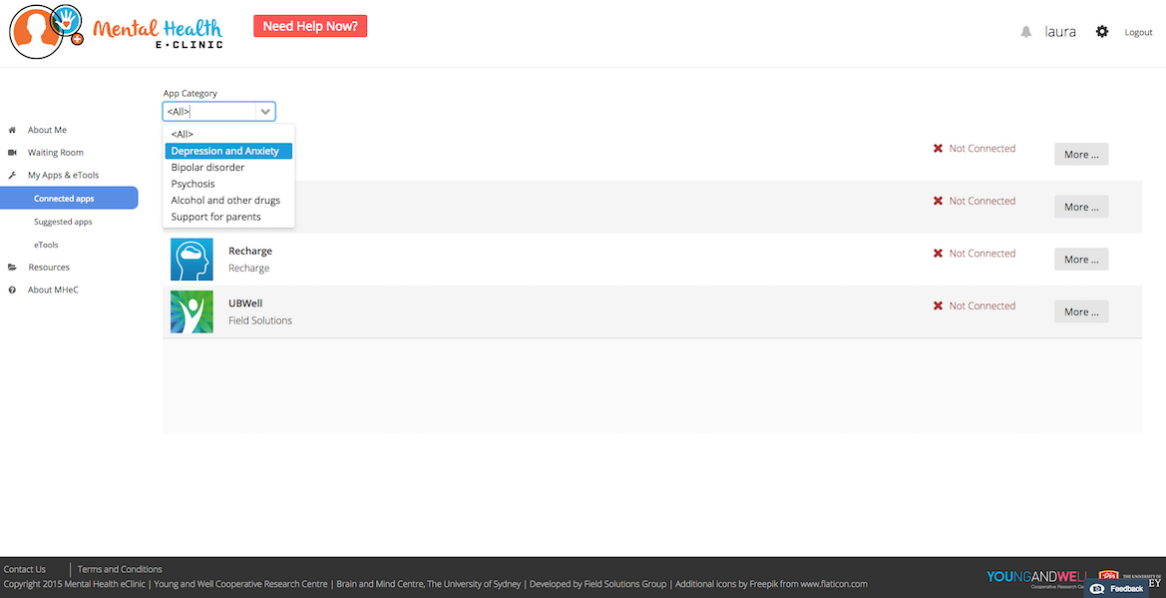
Personalized well-being plan, including recommended apps and etools.

The iterative R&D cycle revealed the importance of five main components of the MHeC. These informed the alpha build of the MHeC (Figure 4-8): a welcoming home page with a visible triage system; a comprehensive physical and mental health assessment; a detailed dashboard of results; a booking and a video visit system; and the generation of a personalized well-being plan that includes links to evidence-based, and health professional-recommended, apps and etools. The five components will have different functionalities depending on whether the user is a young person or a health professional.

#### Element 1: Home Page and Triage

Young people suggested the home page should be a “welcoming space,” where young people can feel “comfortable,” without compromising the authenticity and professionalism of the site. As such they indicated a banner with the institutional logos should be visible at all times at the footer of the home page. Young people also wanted the following features to be included on the home page: testimonials from young people about their experiences with the MHeC (“a young person explaining why they are there and how to use it”); reliable mental health information (resources); information about how to help a friend; frequently asked questions; and a brief explanation about how the MHeC works. Young people also suggested that as this information would be of interest to a wide range of people, the home page needs to be available to everyone, even if they have not signed up for the MHeC. Youth health professionals and young people suggested that, before any login process, a triage system needs to be in place to ensure that young people in distress can access immediate care and a “need help now” button should be clearly visible for those in crisis.

#### Element 2: Online Physical and Mental Health Assessment

Young people said they felt comfortable about completing an online physical and mental health assessment and receiving immediate feedback of their results. The majority also initially reported that they preferred short questionnaires (approximately 15 min in duration). After an explanation was provided, however, about the number and types of questionnaires that would be included to fully assess their physical and mental health (including the range of standardized assessments that are currently being used in *headspace* centers and completed using paper and pencil or iPad) and that a comprehensive assessment would enable a detailed dashboard of results and well-being plan to be generated (including recommended apps and etools), young people understood the need to complete a more comprehensive assessment (up to 1-hour duration). Young people suggested that a pause and “resume later” feature would be helpful with a longer questionnaire to ensure completion. In addition, young people reported that they preferred Likert-scale questions (typically a 5-, 7-, or 9-point agreement scale), two-way close-ended question (no/yes) or multiple-choice questions rather than free-response (“enter text”) questions. The MHeC’s assessment includes medical history, physical health, mental health symptomatology, and health behaviors. For youth health professionals, the questionnaire functionality enables them to write notes or complete/add additional information for relevant questions in relation to their young person’s progress or specific clinical observations (eg, provide a score in the Social and Occupational Functioning Assessment Scale [74] or allocate participants to a clinical stage [47]).

#### Element 3: Dashboard of Results and Progress

Young people reported that after completing the assessment, immediate feedback about their results was essential. They also reported that knowing this would occur would improve motivation to respond as best as possible and answer all questions. Young people said they wanted accurate feedback about their results and for this to be represented in a detailed dashboard (“visual concept”), with the option of printing or saving the file as a PDF for their records, or to share with a health professional in the future. Simple bar graphs, colored icons, and traffic light representations were seen as the most acceptable and understandable options for the presentation of results. Line graphs were the preferred option to represent changes over time and to track progress. Young people also suggested the availability of a “customize option” would be useful to enable individuals to select multiple variables of interest to explore if, and how, they influence another. Youth health professionals would have access to a young person’s results and progress as they reported that a dashboard of results would help guide care before and during video and face-to-face visits.

#### Element 4: Waiting Room, Booking System, and Video Visit System

Young people requested that a booking system be available to make timely appointments with youth health professionals attached to the clinic. Young people also suggested a “waiting room” be included in the MHeC where individuals wait for their video visit to begin. While on this page, young people suggested various activities could be available, for example, breathing exercises or watching selected videos until the video visit icon changes color, signaling their video visit is about to commence. In relation to the youth health professionals’ functionality of this component, they suggested that it would be important to have access to a booking system (“to add or cancel a booking as well as block timeslots”).

Acknowledging cultural preferences, and that some people do not feel comfortable seeing their image (“seeing myself distracts me, I would be looking at myself”), it was suggested by some young people that at the moment of booking an appointment the option of hiding their image should be available. It was also suggested that in the event of communication cut-offs or a young person wanting to say something sensitive, a chat box would allow a fluid conversation. All participants agreed that the video visit system needs to be embedded in the MHeC as “it would be secure.” As it happens in regular practice, the youth health professionals are in charge of inviting a young person to come to their office; therefore, in the MHeC, the youth health professional has the possibility to start and end the video visit. Importantly, this video visit system includes a “share” functionality, where relevant information can be shared and made visible on a young person’s screen (eg, “explain their dashboard of results during the session”).

#### Element 5: Personalized Well-Being Plan That Includes Links to Evidence-Based, and Health Professional–Recommended, Apps and Etools

All participants reported that they would like to be provided with a personalized well-being plan (generated from their assessment results) that included tailored automatic recommendations on how to improve their health and well-being. Young people believe that apps and etools targeting their main concerns/issues would be beneficial. However, young people believe that “health professional recommended” and free apps/etools are more likely to be downloaded over apps/etools that have to be purchased or that are not recommended by health professionals. The areas of main interest to young people were apps/etools that help them with their sleep; improve their memory; assist with mood tracking; and help with tracking their progress over time. Ideally, apps and wearable devices should be integrated with the MHeC. Youth health professionals felt confident about the MHeC suggesting apps and etools to young people “only if they are from a reputable source” and had greater confidence in those that had been independently rated using the Mobile App Rating Scale [75]. In terms of functionality, all end users suggested that the MHeC should have a list of apps sorted by categories such as sleep, mood, anxiety, and physical health, among others. Participants suggested that each app should have an Apple Store and Google Play link to facilitate the download process.

### User Interface

In relation to the look and feel of the MHeC, young people and youth health professionals agree that the system should be “clean and tidy,” displaying short and concise information and making good use of space. Most of the participants preferred to have icons instead of text and preferred to self-explore the system rather than having lengthy instructions. The use of stereotyped photos with “happy” or “sad people” was highly discouraged by young people. The participants wanted to see videos about how to use the system, but indicated that these should be short (no more than 30 s) and not auto-play due to data download size concerns and privacy issues (eg, a video commencing while sitting in a public space). Soothing colors were preferred for the background and brighter colors for the functionalities, suggesting combinations such as blue and orange for consideration. Young people also suggested the possibility of a “customizing” option to enable users to choose between two to three text types and background color options.

### Privacy and Data Sharing

Young males were more concerned about privacy issues than young females. For example, young male participants said “sharing data is OK, but it must say it's confidential” or “I wouldn’t share my location with the system.” On the other hand, young female participants said, for example, “I have no privacy concerns specifically if going to a clinician.” All young people, however, said they would share their data as they believe the MHeC would be a professional and trustworthy site and this would enable them “to get most help.” Young people, however, emphasized that they want to be informed about how the data will be handled before giving permission for data sharing and to have the opportunity to withdraw this permission at any time.

### Integration With Social Networks

Young people believe that the MHeC should be part of social networks as they would like to be able to share the MHeC in their profile or with a friend through a private message. Moreover, they wanted to be able to like the MHeC or write a comment on it. One of the suggested preferred features of the MHeC was “tips” and for these to be displayed throughout all components of the MHeC. Young people also thought it would be valuable if they were able to share these tips via their social networks.

### Usability

Information about the efficiency and effectiveness of the alpha build of the MHeC was obtained during the one-on-one consultation sessions. All participants (n=6) completed usability tasks. All participants found that “creating a MHeC account” and “login” were relatively simple processes. Half of the participants thought that giving the option of login with their social media details (single sign on) was a good idea as it would speed up this process (“super easy”). The other half were against social media login as they were concerned about the MHeC sharing information with their friends (“I don’t want my friends seeing this information”). Finding the “need help now” button was a simple task; all participants found it in less than 5 s. Booking an appointment was a straightforward task as well, and participants were able to navigate the MHeC and complete the process almost immediately.

## Discussion

### Principal Findings

Our study utilized an innovative approach to the development of a Web-based mental health clinic for young people (the MHeC). The PD employed ensured that end users (young people and youth health professionals) had an active and equal involvement in all phases of the design and development process. By engaging these stakeholders, we attempted to respond to end users’ expectations about the MHeC and what was achievable in terms of technology and what has been proven to be effective in the mental health field. The use of several design activities (propositions, hand-drawn sketches, and mockups) and the combination of different PD methodologies (workshops and one-on-one consultations with end users, including assessing usability of the online alpha build of the MHeC) enhanced feedback processes and the generation of new ideas. This combination of research methodologies also accelerated the refinement of the MHeC to achieve the build of the alpha prototype.

In the past decades, the development and use of eHealth solutions in mental health care have expanded; however, these solutions have been developed to address specific problems or to replace different components of the traditional health care system. As an example, the majority of self-triage tools rely on people actively searching for these tools on the Internet; however, some health services provide self-triage tools on their websites, particularly when booking appointments online [26]. Telepsychiatry (videoconferencing), as another example, has also been a particularly effective way of providing support as it allows real-time interaction while negating barriers such as cost, geographical location, and stigma concerns associated with face-to-face support [46]. To the best of our knowledge, our MHeC is one of a kind as it integrates triage, online assessments, online provision of results with easy-to-interpret graphic representations (a dashboard), enables video visits with youth health professionals, provides a personalized well-being plan with immediate interventions, and tracks progress. We believe this is more sophisticated and technologically advanced than traditional telemedicine.

One of the strengths of our system is that it integrates new and emerging technologies with the traditional face-to-face process. Several studies emphasize that screening alone is insufficient for connecting end users with the necessary resources for effective treatment [9,76-78]. Instead, it is argued that online screening should supplement, and be integrated when necessary, with additional support and assessment, which can include face-to-face or online assessments with health professionals within mental health services [9,78]. In line with these recommendations, Internet-based clinical assessments have been implemented and trialed in various Web-based clinics as a means of rapid assessment and referral to appropriate online interventions [79,80] and integrated with face-to-face and online support [20].

Important considerations need to be made in relation to an end user’s health literacy as it is argued that Web-based health care communication demands a higher level of health literacy from end users, including the knowledge and skills that enable them to navigate the health system [81]. National datasets report that 60% of Australians have health literacy scores at the lowest two levels of proficiency (scoring 1 or 2), whereas only 6% attain the highest two levels (scoring 4 or 5) [82]. Furthermore, if an end user is experiencing active psychosis or is in crisis, for example, the ability to accurately read or reflect upon information may prove challenging [83,84].

Video visits (as provided in the MHeC with “share” functionality), and face-to-face support provided by health professionals, may provide end users with the support they need to help them navigate the online physical and mental health assessment, dashboard of results, and the health system. These noted challenges highlight the need for health professionals to remain involved in some capacity in the online assessment process, particularly to prevent the unnecessary funneling of end users into the health system.

In 2017, it was estimated 95% of young people in Australia had access to a mobile phone [85,86], making Internet access more widely available to individuals. Mobile phones also provide individuals with higher levels of privacy (compared with shared computers) when participating in a video visit or completing assessments. Consequently, these devices could serve as great facilitators of health care provision, playing a crucial role in health reform.

### Implications

In 2014, the Australian Government asked the National Mental Health Commission (NMHC) to review existing mental health services and programs and “…assess the efficiency and effectiveness of programs and services in supporting individuals experiencing mental ill health and their families and other support people to lead a contributing life and to engage productively in the community” [87]. Similar to previous reviews, the NMHC, called for an overhaul of the Australian mental health system, including an integration of e-mental health with face-to-face services. Innovations in the use of technology during the assessment process hold potential as they can go some way to addressing documented youth service capacity issues [84,88].

In alignment with the NMHC’s three key components (person-centered design principles; new system architecture; and shifting funding to more efficient and effective “upstream” of services and supports), we envisage that the MHeC will result in a usable system, with high engagement rates, and could finally improve access for all young people requiring assistance across Australia. We see the MHeC as a real-time primary care clinic integrated with current face-to-face services, offering end users at a minimum immediate online clinical assessment; immediate generation of a dashboard of results and personalized well-being plan with tailored interventions; and inbuilt triage and escalation protocols to accurately respond to severe and risky cases. The MHeC offers timely support and interventions through the development of an individual and health professional shared treatment plan. It also provides an opportunity for young people to have a video visit with a health professional despite their current geographical location. Although developed for Australian settings, the MHeC has the potential to be customized for use in developed and developing countries (and especially in those countries where Internet connectivity is high).

Information obtained from this alpha build (more specifically it’s screening and triage protocols; online physical and mental health assessment, video visit system; and self-directed mental health support features) are now informing the development and build of the Synergy Online System (Synergy). Synergy is a Web-based modular platform that links integrated and interoperable resources (eg, apps, etools, data sharing, and access to online and in-clinic health services). Synergy operates through existing health providers to promote access to high-quality and cost-effective mental health services. This system does not deliver services or compete with existing service providers; rather, it aims to complement them by linking with other services. In addition, it enables real-time health and social outcomes tracking, thus providing high-quality and personalized service recommendations to the person seeking help. Synergy has been configured to permit the transfer of individual-level data (allowing for other ethical, consent, governance, and privacy considerations) between it and other existing record systems. The actual capacity to do this in any specific service setting depends on the ways in which existing service systems are configured and governed. Synergy aims to enable Australian mental health service transformation for better outcomes for individual users, their families and supportive others, health professionals, and service providers.

Currently, a series of collaborative research trials are planned to evaluate the use of Synergy across the lifespan. This research is funded by a 3-year agreement (2017-20) between the Australian Government Department of Health and InnoWell Pty Ltd (a joint venture between The University of Sydney and PricewaterhouseCoopers) to the value of Aus $30 M. Results from this research will be reported separately.

### Limitations

Our sample size in Phase 3 (one-on-one consultation with end users) was in the lower range of the average numbers for this type of study (between 6 and 12 participants) [89]. However, our sample size still enabled us to collect sufficient information for analysis in the framework and reach a saturation point. Further research is needed to understand the acceptability and usability of the system, as well as to validate all the components in real-world settings.

### Conclusions

The MHeC has been designed to be accessible, affordable, available, and applicable to the many and varied settings in which there is the opportunity to connect with young people experiencing mental health concerns at anytime, anywhere across Australia, regardless of geographic location. The MHeC especially represents a solution for regional and rural/remote locations and the developing world where the need for mental health care far outstrips the number of health professionals available.

To the best of our knowledge, it is the first time that a Web-based clinic has been conceptualized and codesigned through an iterative cycle with end users. The close collaboration between all different stakeholders has the potential to effectively increase accessibility to mental health services for young people, the quality of care provided to young people, and also increase engagement with, and usability of, the final product. The main components of this system ensure that young people seeking help can find what they need within one system. The alpha prototype of the MHeC not only provides end users with accurate health information but also provides each person with the opportunity to have a comprehensive clinical (physical and mental health) assessment as well as real-time tailored interventions, including a video visit with an appropriate heath professional. Further research is needed to assess the acceptability of the MHeC as well as its applicability in large-scale studies.

## Abbreviations

KT: knowledge translation
MHeC: Mental Health eClinic
NMHC: National Mental Health Commission
PD: participatory design
R&D: research and development
